# Revisiting the Allosteric Regulation of Sodium Cation on the Binding of Adenosine at the Human A_2A_ Adenosine Receptor: Insights from Supervised Molecular Dynamics (SuMD) Simulations

**DOI:** 10.3390/molecules24152752

**Published:** 2019-07-29

**Authors:** Maicol Bissaro, Giovanni Bolcato, Giuseppe Deganutti, Mattia Sturlese, Stefano Moro

**Affiliations:** 1 Department of Pharmaceutical and Pharmacological Sciences, Molecular Modeling Section (MMS), University of Padova, via Marzolo 5, 35131 Padova, Italy; 2 School of Biological Sciences, University of Essex, Wivenhoe Park, Colchester CO4 3SQ, UK

**Keywords:** Adenosine Receptor, Agonist, Sodium Ion, Allosteric Modulator, Molecular Dynamics, Supervised Molecular Dynamics

## Abstract

One of the most intriguing findings highlighted from G protein-coupled receptor (GPCR) crystallography is the presence, in many members of class A, of a partially hydrated sodium ion in the middle of the seven transmembrane helices (7TM) bundle. In particular, the human adenosine A_2A_ receptor (A_2A_ AR) is the first GPCR in which a monovalent sodium ion was crystallized in a distal site from the canonical orthosteric one, corroborating, from a structural point of view, its role as a negative allosteric modulator. However, the molecular mechanism by which the sodium ion influences the recognition of the A_2A_ AR agonists is not yet fully understood. In this study, the supervised molecular dynamics (SuMD) technique was exploited to analyse the sodium ion recognition mechanism and how its presence influences the binding of the endogenous agonist adenosine. Due to a higher degree of flexibility of the receptor extracellular (EC) vestibule, we propose the sodium-bound A_2A_ AR as less efficient in stabilizing the adenosine during the different steps of binding.

## 1. Introduction

The human genome encodes more than 800 different G protein-coupled receptors (GPCRs), membrane proteins characterized by a distinctive seven transmembrane helices (7TM) architecture. This superfamily of receptors recognizes an enormous variety of extracellular signals (i.e., ions, neurotransmitters, peptides) and transmits the chemical information into the intracellular compartment, modulating many cellular activities [1,2,3]. This is achieved through the recruitment of different molecular effectors, such as G proteins, protein kinases, or β-arrestins. Given their crucial role at the cellular level, GPCRs represent an important family of therapeutic targets, and it is not surprising that more than 30% of the approved drugs act on at least one GPCR [2].

Adenosine receptors (ARs) are a family of class A GPCRs comprising four different subtypes, respectively, A_1_, A_2A_, A_2B_, and A_3_, all involved in purinergic signaling [3]. ARs recognize the extracellular nucleoside adenosine as the endogenous agonist, which, depending on the receptor subtype and tissue localization, affects and modulates different pathophysiological cellular conditions in a pleiotropic way. For example, purinergic signalling is involved in inflammation, cancer, neurodegeneration, and cardiovascular diseases [4]. The human A_2A_ AR subtype has been studied in depth both from a pharmacological and structural point of view. To date, 46 structures deposited in the Protein Data Bank (PDB) show the adenosine A_2A_ receptor (A_2A_ AR) in complex with both agonists (active and intermediate active states) and antagonists (inactive states) [5].

Interestingly, the A_2A_ AR was the first GPCR co-crystallised with a monovalent sodium ion, explaining from a structural point of view its negative allosteric effect [6]. In 1973, Pert and co-workers discovered how physiological concentrations of specific ions could decrease the opioid receptor affinity for agonists, without influencing the antagonist’s binding profile [7,8]. After this first body of evidence, the effect of the sodium ion (Na^+^) was particularly investigated, leading to the discovery of at least 15 further GPCR subtypes sensible to its allosteric effect. Site-directed mutagenesis studies led to the identification of the conserved amino acid D^2.50^ as a fundamental counterpart for sodium binding, later confirmed by the publication (2012) of the first high-resolution (1.8 Å) X-ray crystal structure of the A_2A_ AR [6,9]. In this structure, the Na^+^ was located at the interface between TM2, TM3, and TM7, coordinated to five oxygen atoms belonging to the side chain of the conserved residues D^2.50^, S^3.39^ (the Ballesteros-Weinstein GPCRSs numbering is reported as superscript) and to an ordinate cluster of three water molecules. The negatively charged aspartic acid is conserved in over 90% of the class A GPCRs, thus suggesting an evolutive role in binding the monovalent ion [10,11,12]. As reported in Table 1, 34 GPCRs have been co-crystallized with a sodium ion, spanning members from three of the four branches in which the class A GPCRs are classified.

A large body of structural evidence indicates that the sodium ion is detectable exclusively in the presence of antagonists, as all the GPCRs solved in the active state do not coordinate the cation. It follows that a receptor can exist in at least two conformational states, one able to bind the sodium ion and antagonists, the other with high affinity only for agonists. From a functional point of view, it has been proposed that the sodium stabilizes a specific conformation of the receptor and shifts the conformational equilibrium towards the inactive state [13]. In light of this, computational studies turned their attention to the influence of sodium ion coordination in the A_2A_ AR affinity for antagonists, focusing less on the structural basis of the sodium-bound receptor’s inability to recognize agonists [14]. The sodium binding mechanism to 18 different GPCRs has been recently investigated through microsecond-scale molecular dynamics (MD) simulations [15]. Previous computational studies compared the allosteric binding site of the sodium ion in the A_2A_ AR inactive and active states, suggesting the latter conformation is characterized by an important reduction of the volume of the allosteric cavity, unfavorable to the ion coordination [9,16]. Although it is now widely accepted that the recognition of the sodium ion at its allosteric binding site occurs from the extracellular side, it is more complex to computationally describe how the sodium may dissociate and how the agonist can play a role in this process [15]. Recent scientific work has shown that Na^+^ can leave the allosteric site either by translocating in the cytoplasmic side or by retracing the binding path towards the extracellular environment. Moreover, the protomeric state of the titratable residue D^2.50^ seems to be determinant in controlling the Na^+^ unbinding mechanism [17,18,19,20]. Further studies are therefore necessary to investigate, from a mechanistic point of view, the negative allosteric modulation of the sodium ion and attempt to understand how the stabilization of the inactive state of the receptor results, from a macroscopic point of view, in a decreased ability of the receptor to recognize an agonist.

In our laboratory we have implemented a computational method, named supervised molecular dynamics (SuMD), that enables the exploration of ligand-receptor recognition pathways in the nanosecond timescale [21,22,23]. The performance speedup is due to the combination of a tabu-like supervision algorithm on the ligand-receptor distance with classical MD simulation. SuMD enables the investigation of binding events independently from the ligand starting position, its chemical structure (small molecules or peptides), and the thermodynamic affinity [21,22,23]. In this work, we simulated and analysed the recognition between the sodium ion and the A_2A_ AR, both in the inactive and intermediate-active conformations. SuMD simulations shed light on the molecular basis underneath the allosteric effect of the sodium ion from a site distinct from the orthosteric one, allowing for a better understanding of how its presence perturbs the binding mechanism of the endogenous agonist adenosine.

## 2. Results and Discussion

### 2.1. SuMD Simulations of the Sodium Ion on the A_2A_ AR

As anticipated, SuMD simulations allow for the simulation of intermolecular recognition pathways in a very compressed time scale. However, this limits exploration to a limited subset of the complex GPCR conformational landscape during a single SuMD simulation. Considering also the lack of reliable structural information on the unbound (apo) state of the receptor, the experimentally-determined inactive (co-crystallised with the inverse agonist ZM241385) and intermediate active (co-crystallised with the adenosine) conformations of A_2A_ AR were retrieved from the PDB database (PDB codes: 4EIY and 2YDO, respectively) and prepared for the SuMD simulations, as described in the Materials and Methods section. In order to ensure the robustness of the results, five SuMD replicates for each state of the receptor were performed to simulate the recognition of the sodium ion. As far as we know, this is an expansion of the applicability domain of this MD method; previously it was only regarding small molecules and peptides. As reported in Table 2, a few nanoseconds were sufficient to sample a complete Na^+^ binding pathway during each repetition, instead of several microseconds as required by classical MD experiments [15].

**Figure 1:**
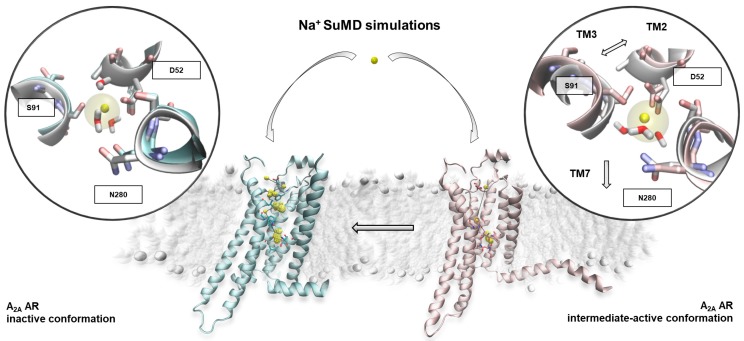
The recognition pathway of Na^+^ on the two relevant A_2A_ AR conformations. TM = transmembrane helices.

On the inactive A_2A_ AR conformation, the cation reached the allosteric site (identified by the triad of residues D^2.50^, S^3.39^, and N^7.45^) in four out of five SuMD replicates (low RMSD_min_ values in Table 2), reproducing the experimental coordination with three water molecules (Figure 1, Video 1).

Surprisingly, the sodium ion also reached the allosteric binding site during three out of the five SuMD replicates of the receptor intermediate-active conformation, which has been suggested as the low-affinity state for the cation. In line with the results from a previous study, the active conformation of A_2A_ AR was able to bind the sodium only after a rearrangement of the TM domain (TMD), characterized by the increase of the distance between the TM2 and TM3, as well as the outward movement of the TM7 (Figure 1) [16]. Of note, these are hallmarks of the inactivation process of GPCRs [24].

On the left side of the panel in Figure 1, the inactive state of the receptor is reported, along with the sodium positions mainly occupied during the SuMD replicates (yellow dots). The ten most engaged residues are shown as a stick. Within the round box, a magnification shows the sodium allosteric site from a SuMD representative frame (cyan ribbon) and the crystallographic reference 4EIY (white ribbon). The cation reached the experimentally-solved position (transparent van der Waals volume). On the right side, the intermediate active conformation of the A_2A_ AR is reported alongside the ion positions during binding (yellow dots). A SuMD final state (pink ribbon) and the crystallographic reference 2YDO (white ribbon) are compared in the magnification. The corresponding sodium location in the inactive structure 4EIY is shown as a transparent van der Waals volume. The receptor’s structural changes upon sodium binding (indicated with arrows) can be summarised with an increase of the inter-helical distances in order to accommodate the cation.

To better analyse the sodium ion recognition against the two A_2A_ AR conformations, the SuMD trajectories were subjected to a clustering analysis using the DBSCAN algorithm (for details see the Materials and Methods section), which was able to geometrically map the regions of the receptor in which the cation was stationed the most during its approach to the allosteric site (Figure 1, Figure S8) [25]. The clusters highlighted a binding mechanism articulated in three temporally consequent phases. During the first step, the sodium ion approached the vestibular region of the A_2A_ AR and interacted with negatively charged residues located at the second extracellular loop (ECL2). A strong electrostatic interaction was formed with E169^ECL2^, before the breaking of the E169^ECL2^–H264^ECL3^ salt bridge [26]. Interestingly, in Replicate 1 (the only unproductive simulation of the active A_2A_ AR) the ion remained trapped in proximity to the ECL2 as strong interactions with E169^ECL2^ was retained for the entire simulation. In the successive binding step, the sodium ion explored the orthosteric site and made interactions with residue N253^6.55^, known to be fundamental for the binding of both agonists and antagonists. The final transition of the sodium to the allosteric site (step three) was controlled by the side-chain rotameric state of the “toggle switch” W246^6.48^ residue [16,27]. Although the sodium binding modes obtained from simulations on the two A_2A_ AR conformations were similar (Figure 1), the recognition mechanism of the sodium ion significantly diverged (Figure S8). On the active A_2A_ AR, indeed, the cation did not situate on the orthosteric site, putatively due to a different conformational state of the W246^6.48^ side chain (which has been suggested as being able to modulate the communication between the orthosteric and allosteric sites ) [28].

To investigate the reversibility of the sodium ion binding to the inactive A_2A_ AR, an unbiased MD simulation was performed from a SuMD replicate’s final state (see the Materials and Methods section). As expected, in about 600 ns, a spontaneous unbinding event from the allosteric site was sampled (Figure S9).

SuMD simulation results suggested that in absence of the orthosteric ligand, the ion could spontaneously coordinate and stabilize the inactive conformation of the receptor (the receptor state also responsible for the antagonists and inverse agonists recognition). On the other hand, Na^+^ was able to bind the active state of the receptor only after an adaptation of the allosteric binding site. Only that conformational population not bound to the sodium ion, in equilibrium with the previous one, could, therefore, be recognized by an agonist, ready to trigger the receptor activation process. In this way, we could give a molecular interpretation to the pharmacological meaning of the negative allosteric modulator attributed to the sodium ion.

To investigate the possible effects that these two different Na^+^–A_2A_ AR complexes can trigger on the binding mechanism of the endogenous agonist, adenosine, further SuMD replicates were carried out and the results will be described in the next sections.

### 2.2. SuMD Simulations of the Adenosine on the Intermediate-Active, Sodium-Free, A_2A_ AR Conformation

Ten SuMD replicates (Table 3) were performed using the A_2A_ AR coordinates in the intermediate-active conformation (PDB ID 2YDO). We define “productive” as a trajectory that resulted in the adenosine reaching the orthosteric site. The seven productive SuMD simulations were extended for a further 100 ns of unbiased MD simulation to evaluate the stability of the bound states sampled.

**Figure 2:**
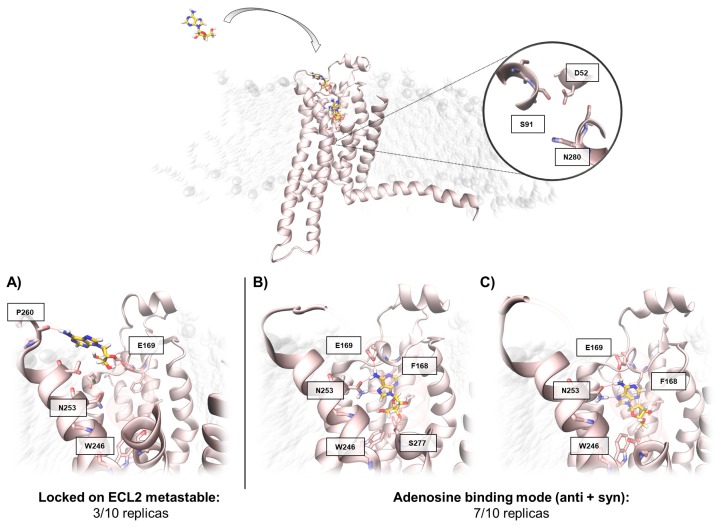
Conformations sampled by the adenosine while recognizing the A_2A_ AR in the intermediate-active state. Top, the absence of a sodium ion in the allosteric binding site is highlighted. Panel (**A**) shows a representative adenosine binding mode in the extracellular loop 2 (ECL2) metastable binding. In panels (**B**,**C**), the ribose in *anti* (**B**) and *syn* (**C**) conformation are reported. Only the *syn* orientation permits the hydrogen bonding with the residue S277^7.42^.

We begin the description of the results from trajectories 1a, 6a, and 10a, in which the adenosine did not reach the orthosteric site (Table 3). Interestingly the ligand extensively sampled a metastable-binding site at the interface between ECL2 and ECL3, putatively representing an ancillary site of recognition besides the orthosteric one [23,29]. This intermediate binding mode was characterized by the polar interaction between the adenosine ribose moiety and the negatively charged residue E169^ECL2^, as well as hydrophobic contacts with M174^5.35^ and transient hydrogen bonds with residues at the ECL3 (Figure 2A). The interaction energy analysis (Figure S3) suggests that the stability of this metastable state is comparable with the adenosine in its crystallographic binding mode (Figure S4) and justifies the missed transition to the orthosteric site.

The seven productive SuMD simulations (Table 3) allowed the adenosine to explore different conformations within the orthosteric site, including the crystallographic one. Trajectory 3a, indeed, was able to reproduce with great accuracy (RMSDmin = 0.45 Å) the experimental binding mode (Video 3), with all the key interactions faithfully recovered (Figure 2B) [30]. Interestingly, trajectories 2a, 4a, and 5a described an alternative recognition mechanism, according to which the adenine ring of the agonist approaches the binding site, orienting the ribose moiety towards the extracellular (EC) receptor vestibule ("ribose-up" conformation) [31,32]. These states were transient, as the classic MD simulations rapidly evolved towards the crystallographic binding mode, but without sampling the key hydrogen bond with residue S277^7.42^ side chain (Figure 2C), due to the so-called *syn* conformation of the β-glycosidic bond (*anti* in the crystal structure).

### 2.3. SuMD Simulations of the Adenosine on the Inactive, Sodium-Bound, A_2A_ AR Conformation

**Figure 3:**
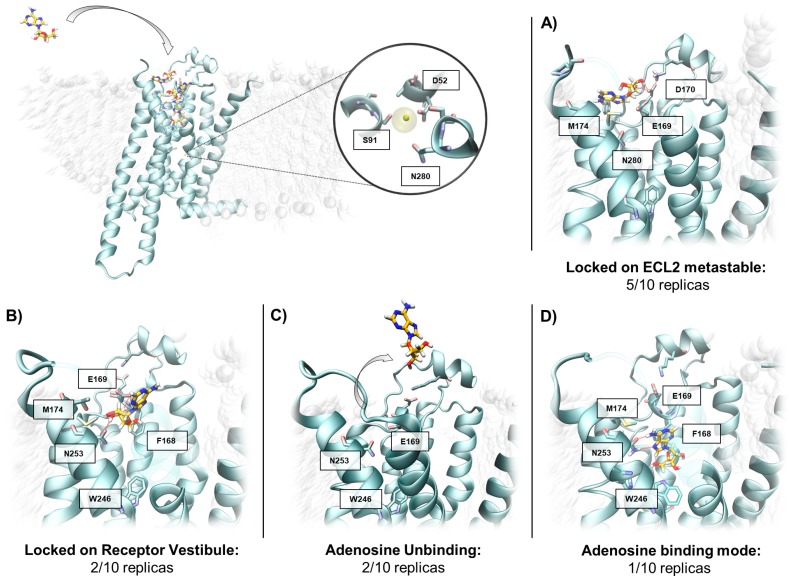
Conformations sampled by the adenosine while recognizing the A_2A_ AR in the inactive state. Top, the presence of the sodium ion in the allosteric binding site is highlighted. In panel (**A**), a representative adenosine binding mode in the ECL2 metastable binding site is depicted. In panel (**B**), one of the different conformations sampled by the adenosine in the receptor vestibule is reported. Panel (**C**) summarizes the number of ligand unbinding events collected, starting from the vestibule region. Panel (**D**) represents the only SuMD simulation (Replica 10i) that showed an adenosine crystallographic binding mode.

As anticipated, to verify the different adenosine propensities to recognize divergent A_2A_ AR conformational states, SuMD was performed on the inactive conformation of the receptor (PDB ID 4EIY), retaining the sodium in its allosteric site (Figure 3) but depleting the inverse agonist ZM241385. Consistently with the first part of this work, ten SuMD replicates were collected (as summarized in Table 4).

Unlike the intermediate-active conformation, on the inactive, sodium-coordinated A_2A_ AR just one replication out of ten resulted in the adenosine reproducing the experimental binding mode. Specifically, in half of the trajectories sampled (replicates 1i, 2i, 6i, 7i, and 8i in Table 4) adenosine did not reach the orthosteric site, but sampled the solvent-exposed metastable binding site at the interface between ECL2 and ECL3 (Figure 3A), again interacting with E169^ECL2^ as reported in the previous section of the manuscript. The remaining five SuMD simulations were instead defined as quasi-productive, since the agonist reached the vestibular region of the orthosteric binding site without, however, reproducing the adenosine crystallographic pose. Lee and collaborators investigated, by means of classical MD simulation, the behaviour of adenosine within the inactive-state A_2A_ AR orthosteric site and pointed out the agonist’s inability to maintain the original binding mode, thus corroborating our SuMD results [33].

To evaluate the stability of the five quasi-productive SuMD final states (replicas 3i, 4i, 5i, 9i, and 10i) the trajectories were prolonged for 100 ns (unbiased MD). As reported in Figure 3B, during the extended trajectories, 3i and 5i the adenosine maintained its vestibular position. Trajectories 4i and 9i, on the other hand, were characterized by the spontaneous dissociation of the ligand, indicating a poor ligand stabilization (Figure 3C). Curiously, the extended trajectory of 10i was the only one during which the adenosine reached the experimental bound state (RMSD_min_ = 0.3 Å Table 4, Figure 3D).

### 2.4. Insight on the Role of the Sodium Ion in the Recognition of A_2A_ AR Agonists

In a schematic way, Na^+^ coordination within the allosteric TMD allows for the discrimination of the two main conformational states of A_2A_ AR (i.e., active and inactive); it is capable of recognizing adenosine with antithetical efficiency, as suggested by the divergent binding frequencies sampled through the SuMD simulations. As highlighted in Figure S10, in the supplementary material, the limited structural differences between the two crystallographic conformations of the receptor would not be sufficient to explain, from a mechanistic point of view, the negative allosteric effect mediated by a sodium ion. Consequently, the use of techniques able to take into consideration the conformational plasticity associated with the receptor functionality is found to be essential to realistically rationalize the role played by the monovalent ion.

**Figure 4:**
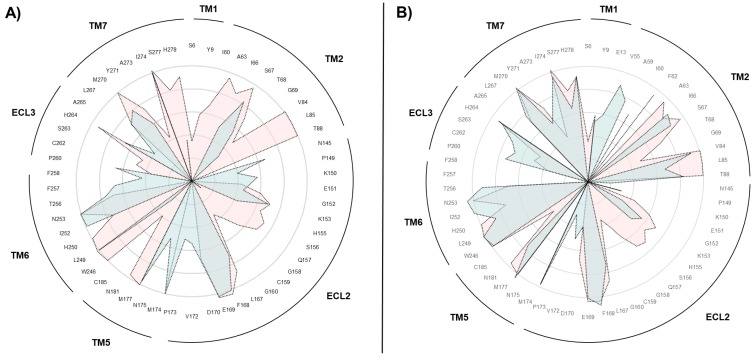
The adenosine experienced different patterns of interactions during SuMD dynamic docking on the intermediate-active and inactive A_2A_ AR conformations. The adenosine-A_2A_ AR contacts are plotted as polar diagrams of overlapping data. In panel (**A**) replicate 3a (productive binding to the intermediate-active receptor state, pink) and Replicate 5i (quasi-productive to the inactive receptor state, cyan) are compared. In panel (**B**), replicate 3a (productive binding to the intermediate-active receptor state, pink) is compared with replicate 10i (the only productive binding to the inactive receptor state, cyan).

To decipher the molecular basis underneath such misleading outcomes described by the SuMD simulations (i.e., replicas 3a and 5i, sampled, respectively, starting from the active and inactive receptor states), cumulative maps of the interatomic contacts between adenosine and A_2A_ AR binding site residues were graphically depicted, using polar diagrams. As reported in Figure 4, box A, the agonist’s inability to reproduce the canonical experimental conformation in the receptor inactive state is accompanied by discrepancies in the adenosine recognition pathway, mainly at the level of TM1, TM2, and TM7. These differences, on the other hand, were not noticed during replicate 10i, the only productive trajectory sampled starting from the inactive state of the receptor in the presence of the sodium ion, as indicated in Figure 4, box B. These data further emphasize the importance of residues located in TM1, TM2, and TM7 for the correct molecular recognition process of agonists.

Deciphering the dynamics of the A_2A_ AR states is fundamental to interpreting the discrepant agonist recognition pathways. In a recent computational investigation, increased flexibility of A_2A_ AR EC domains was described in the receptor inactive state, a phenomenon that is less relevant in the active conformation and thus could help in differentiating agonist binding mechanisms [33]. To verify if this evidence can be extrapolated from our SuMD simulations, the volume of the orthosteric binding site was dynamically monitored in the two aforementioned trajectories (replicates 3a and 5i). Interestingly, even if the starting volumes computed for the A_2A_ AR binding site on both crystallographic structures taken under examination were quite similar (~250 Å^3^), only a few ns of the simulation were required to reveal the different evolutions of the two systems.

**Figure 5:**
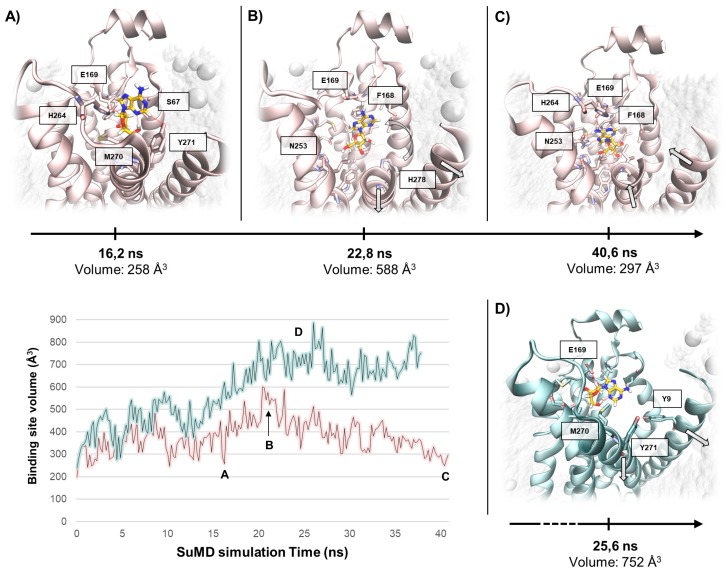
The orthosteric site volumes change differently during SuMD simulations of the intermediate-active and the inactive A_2A_ ARs. Panels (**A**–**C**) depict three snapshots from SuMD Replicate 3a, related to the key steps of the adenosine recognition. Initially, the agonist approaches the A_2A_ AR extracellular vestibule (**A**) and through a polar interactions network mediated by ECL3, TM2, and TM7 (whose overall organization is not perturbed with respect to the crystal structure), inserts the purine ring into the binding site (**B**). The adenosine is then able to reach the canonical binding mode (**C**) only when the cavity volume recedes toward the original value. On the A_2A_ AR inactive state, the binding site volume progressively increases due to the TM1 and TM7 outward movements (Panel (**D**)), making the agonist binding more difficult.

On the intermediate-active conformation of the A_2A_ AR, adenosine approached the receptor, interacting with the vestibular region ECL2 (Figure 2A). The transition to the orthosteric binding site was mediated by a series of polar interactions with residues located at the ECL2, TM2, and TM7. In this phase, the compactness of the receptor orthosteric site was necessary for the productive adenosine recognitions, as indicated by the small fluctuation of the cavity volume (Figure 5A). From this standpoint, the accommodation of the adenosine in the orthosteric site required the first adaptation of the surrounding TM helices, as suggested by a transient increase in the volume up to a value of about 600 Å^3^ (Figure 5B). Subsequently, the π-stabilizing interaction of the adenine nucleus with the side chain of Phe168 compacts the structure of the recognition cavity, bringing its volume back to a value similar to the initial one (Figure 5).

The presence of the sodium ion within its putative binding site in the inactive A_2A_ AR conformation markedly altered the receptor flexibility. Indeed, during the first step of the simulation, the TM1 and TM7 moved outwards, progressively increasing the volume of the orthosteric site up to about 700 Å^3^, not allowing the driving interactions to the bound final state to be established (Figure 4D). As previously described, the outward movement of segment TM7, combined with TM2 shifting from TM3, represents the key steps for Na^+^ coordination in the active state of A_2A_ AR. It is reasonable to speculate that the presence of the monovalent ion in the middle of the 7TM bundle could be responsible for the greater flexibility of the extracellular portion of the receptor, allowing it to alter the dynamics of the TM2 and TM7, and thus the agonist binding mechanism.

## 3. Materials and Methods

### 3.1. General

MOE suite (Molecular Operating Environment, version 2018.0101) was exploited to perform most of the general molecular modelling operations, such as proteins and ligands preparation [34]. All these operations have been performed on an 8 CPU (Intel® Xeon® CPU E5-1620 3.50 GHz) Linux workstation. Molecular dynamics (MD) simulations were performed with an ACEMD engine on a GPU cluster composed of 18 NVIDIA drivers, whose models go from GTX 780 to Titan V [35]. For all the simulations, the CHARMM36/CHARMM general force field (CGenFF) combination was adopted [36,37,38].

### 3.2. Systems Preparation

Agonist and antagonist-bound complexes of A_2A_ AR were retrieved from the RCSB Protein Data Bank database (PDB ID 2YDO and 4EIY respectively) and handled by means of the MOE protein structure preparation tool [6,30]. Hydrogen atoms were assigned according to Protonate-3D, and any missing loop was modelled with the homology modelling protocol [39]. In the case of PDB ID 4EIY, the apocytochrome b562 (BRIL) inserted in the ICL3 was removed prior to protein preparation and subsequent loop modeling. Missing atoms in the side chains, as well as non-natural N-terminals and C-terminals, were rebuilt according to the CHARMM force field topology [36]. A_2A_ AR apo forms were obtained by simply deleting the orthosteric ligands from their respective complexes. Adenosine force field parameters were retrieved from the Paramchem web service, in concordance with CGenFF [37,38].

### 3.3. Solvated System Setup and Equilibration

Systems were embedded in a 1-palmitoyl-2oleyl-sn-glycerol-3-phospho-choline (POPC) lipid bilayer, according to the pre-orientation provided by the Orientations of Proteins in Membrane (OPM) database and by using the VMD membrane builder plugin [40,41]. Lipids within 0.6 Å from the protein were removed and TIP3P model water molecules were added to solvate the system by means of Solvate1.0 [42,43]. System charge neutrality was reached by adding 100 Na^+^ atoms and 111 Cl^+^ counterions to a final concentration of 0.154 M (A_2A_ AR net charge was +11 for both the system-simulated 2YDO/4EIY). Equilibration was performed through a three-step procedure. In the first step, 1500 conjugate-gradient minimization steps were applied to reduce the clashes between proteins and lipids. Then, a 5 ns long MD simulation was performed in the NPT ensemble, with a positional constraint of 1 kcal mol^−1^ Å^−2^ on ligand, protein, and lipid phosphorus atoms. During the second stage, 10 ns of MD simulation in the NPT ensemble were performed constraining all the protein and ligand atoms but leaving POPC residues free to diffuse in the bilayer. In the last equilibration stage, positional constraints were applied only to the ligand and protein backbone alpha carbons for a further 5 ns of MD simulation.

All the MD simulations were performed using the following protocols: an integration time step of 2 fs; a Berendsen barostat maintained the system pressure at 1 atm; a Langevin thermostat maintained the temperature at 310 K with a low dumping of 1 ps^−1^; the M-SHAKE algorithm constrained the bond lengths involving hydrogen atoms [44,45,46].

### 3.4. Supervised Molecular Dynamics (SuMD) Simulations

Supervised molecular dynamics (SuMD) simulations were exploited to sample and characterize the binding pathway of the Na^+^ monovalent ion, as well to simulate the binding of the endogenous agonist adenosine to the two pharmacologically relevant A_2A_ AR conformations [21,22,23,31]. SuMD methodology reduces the timescale necessary to sample a binding event in the range of nanoseconds, instead of hundreds of nanoseconds or microseconds usually necessary with unbiased MD. Sampling is improved by applying a tabu-like algorithm that monitors the distance between the ligand and center of mass of the protein binding site, during unbiased MD simulations. A series of short unbiased MD simulations are performed, and after each simulation, the distance points collected at regular time intervals are fitted into a linear function. Only productive MD steps are maintained, those in which the computed slope is negative, indicating a ligand approach to the binding site. Otherwise, the simulation is restarted by randomly assigning the atomic velocities. The length of each SuMD step in which the supervision is carried out was adapted relative to the nature of the ligand under investigation. In terms of the sodium ion, given its important diffusion rate, a 200 ps SuMD time window proved to be adequate to accurately describe the binding, whereas for adenosine, the classic SuMD time window of 600 ps, previously optimized and validated for small organic molecules, was set. Short simulations are perpetuated under supervision until the distance between the ligand and the binding site dropped below 5 Å, then the supervision was disabled, and a classical MD simulation was performed. In the present study, for the computation of the allosteric Na^+^ binding site center of mass, residues D52, S91, and N280 were chosen; for the orthosteric A_2A_ AR binding site, residues N253, F168, H250, and H278 were selected.

In all SuMD productive replicates in which adenosine reached the orthosteric binding site, the final state evolution and stability was evaluated through the collection of a 100 ns long classical MD.

### 3.5. SuMD Trajectory Analysis

All the SuMD trajectories collected were analysed by an in-house tool written in tcl and python languages, as described in the original publication [22]. Briefly, the dimension of each trajectory was reduced saving MD frames at a 20 ps interval, each trajectory was then superposed on the first-frame Cα carbon atoms of the A_2A_ AR, and wrapped into an image of the system simulated under periodic boundary condition. In those cases where a reference was present, the RMSD of the ion or adenosine molecule was computed with respect to the experimental crystallographic complex (4EIY for sodium and 2YDO for adenosine). The RMSD values were plotted over time and reported in the movies present in the supplementary materials.

SuMD trajectories investigating the recognition pathway of sodium were furthermore geometrically analysed to identify significant populations of ion position, among the multitude of sampled data. Prody, a python framework for MD manipulation and analysis, was exploited to compute the pairwise root mean square deviations (RMSDs) of Na^+^ atomic coordinates, during all replicates collected [47]. From each replicate, a square matrix of RMSDs was obtained (nf x nf), in which nf stands for the number of trajectory frames. Subsequently, DBSCAN, a density-based clustering algorithm, part of the scikit-learn python packages, was applied to cluster the different ion atomic positions and graphically represent them by exploiting VMD software [25,31]. The orthosteric binding site volume was dynamically monitored in the SuMD trajectories of adenosine recognition, collected starting from the two different A_2A_ AR conformations. POVME 2 python software was exploited to perform the calculation, after defining a spherical inclusion region cantered on agonist centroid coordinates and characterized by a 9 Å radius dimension [48].

## 4. Conclusions

The molecular mechanism that triggers the negative allosteric modulation of the sodium ion on the A_2A_ AR agonists is not fully understood. X-ray structural studies have pointed out the presence of a binding site for the cation in the core of the TMD of the resting receptor (and many other class A GPCRs). However, the high degree of similarity with the intermediate-active (agonist-bound) state of the receptor (Figure S10) does not completely clarify the molecular basis of this effect. In this study, the SuMD technique was therefore employed to simulate the binding processes of the sodium ion and the endogenous agonist adenosine on these two different A_2A_ AR conformations (the intermediate-active and inactive one, respectively), in the attempt to retrieve mechanistic insight.

The Na^+^, whose concentration in the extracellular environment is close to 140 mM, has a fundamental role in controlling the conformational landscape of the A_2A_ AR, characterized by few, highly populated, stable states. The most accepted model describes the sodium as capable of selectively binding only to the inactive-like receptor population. The macroscopic effect of this is a shift of the equilibrium towards the resting state of the receptor, and a decrease in affinity towards agonists. In keeping with this conformational selectivity as well with previous work, our simulations outlined the A_2A_ AR inactive structure as able to coordinate the sodium ion without any topological modification of the putative allosteric site.^10,16^ On the other hand, during the simulated binding on the intermediate-active conformation, an increase of the inter-TM distances was necessary to accommodate the cation, possibly anticipating a receptor transition toward the inactive-state. The “toggle switch” W246^6.48^ was pointed out as a possible gatekeeper of the sodium binding event. Interestingly, SuMD suggested different binding paths on the two A_2A_ AR states. It is intriguing to speculate that the inactive state of the receptor could selectively drive the binding of the sodium ion by putatively shaping the charge distribution of the meta-stable binding sites along the path.

During the successive SuMD simulations, the endogenous agonist showed a propensity to bind the sodium-free intermediate-active state of the receptor (Video 2). Indeed, seven simulations out of ten resulted in an orthosteric complex, while only one SuMD replicate on the inactive structure was productive. We propose the different flexibilities of the extracellular side of the receptor (where the first interactions able to influence the agonists binding occur) as a driving force of this divergence. The presence of the sodium ion in its allosteric site possibly prevented the receptor from adapting to the incoming agonist, due to an opening up of the EC vestibule and, in turn, of the orthosteric site. As a partial confirmation of this, the TM1, TM2, and ECL2 formed less extensive contacts with the adenosine in the inactive A_2A_ AR (Figure 4) due to the increased volume of the orthosteric site (Figure 5).

The speculative mechanism proposed in this work should be further investigated on other GPCRs.

## Figures and Tables

**Table 1:** Crystallographic structures of class A G protein-coupled receptors (GPCRs) deposited on the Protein Data Bank (PDB) and containing a sodium ion in the transmembrane helices (TM) region.

	Best Resolution (Å)	Number of Structures	Class A Branch
A_2A_ adenosine receptor	1.7	24	α
Protease-activated receptor 1	2.2	1	δ
Protease-activated receptor 2	2.8	2	δ
β_1_ adrenergic receptor	2.1	3	α
D_4_ dopamine receptor	2.1	1	α
Complement component 5a receptor 1	2.2	1	γ
δ opioid receptor	1.8	2	γ

**Table 2:** Supervised molecular dynamics (SuMD) simulations of the sodium ion performed on the inactive (left side) and intermediate active (right side) conformations of the adenosine A_2A_ receptor (A_2A_ AR). For each replica, the SuMD simulation time, the positive or negative outcome and the minimum RMSD (RMSDmin) reached by the sodium have been reported (the crystallographic structure 4EIY was used as a reference).

	A_2A_ AR Inactive Conformation			A_2A_ AR Intermediate Active Conformation		
	SuMD time (ns)	Reached the allosteric site	RMSD_min_ (Å)	SuMD time (ns)	Reached the allosteric site	RMSD_min_ (Å)
Replica 1	10.8	No	10.03	15.4	Yes	0.2
Replica 2	23.6	Yes	0.17	12.0	Yes	0.1
Replica 3	20.6	Yes	0.04	2.4	No	24.2
Replica 4	20.8	Yes	0.18	15.6	Yes	0.1
Replica 5	18.2	Yes	0.40	4.6	No	17.1

**Table 3:** Summary of the adenosine SuMD simulations performed on the A_2A_ AR intermediate-active conformation. For each replicate, the SuMD simulation time required, the positive or negative outcome, and the binding mode sampled at the end are reported along with the RMSDmin (calculated using 2YDO as a reference). MD = molecular dynamics.

	SuMD Time (ns)	Reached the Orthosteric Site	Adenosine Binding Mode	X-ray Binding Mode after 100 ns of MD	RMSD_min_ (Å)
Replica 1a	7.2	No	No (Meta-binding site on ECL2)	-	14.3
Replica 2a	31.8	Yes	No (Distorted binding mode)	Yes	0.4
Replica 3a	40.8	Yes	Yes	Yes	0.4
Replica 4a	32.4	Yes	No (Ribose Up)	Yes (Ribose *syn* conformation)	2.5
Replica 5a	29.4	Yes	No (Ribose Up)	Yes (Ribose *syn* conformation)	2.7
Replica 6a	15.6	No	No (Meta-binding site on ECL2)	-	12.2
Replica 7a	28.2	Yes	No (Ribose Up)	Yes (Ribose *syn* conformation)	0.4
Replica 8a	32.4	Yes	Yes (Ribose syn conformation)	Yes (Ribose *syn* conformation)	2.7
Replica 9a	24.0	Yes	No (Distorted binding mode)	Yes (Ribose *syn* conformation)	2.3
Replica 10a	10.2	No	No (Distorted binding mode)	-	15.3

**Table 4:** Summary of the adenosine SuMD simulations performed on the inactive conformation of the A_2A_ AR. For each replicate, the SuMD simulation time required, the positive or negative outcome, and the binding mode sampled at the end are reported along with the RMSDmin (calculated using 2YDO as a reference).

	SuMD Time (ns)	Reached the Orthosteric Site	Adenosine BINDING Mode	X-ray Binding Mode after 100 ns of MD	RMSD_min_ (Å)
Replica 1i	9.0	No	No (Meta-binding site on ECL2)	-	16.1
Replica 2i	16.8	No	No (Meta-binding site on ECL2)	-	13.6
Replica 3i	16.2	Yes	No (Receptor Vestibule)	No (Receptor Vestibule)	5.5
Replica 4i	31.2	Yes	No (Receptor Vestibule)	No (Adenosine unbinding)	6.1
Replica 5i	37.8	Yes	No (Receptor Vestibule)	No (Receptor Vestibule)	6.6
Replica 6i	24.6	No	No (Meta-binding site on ECL2	-	15.4
Replica 7i	7.8	No	No (Meta-binding site on ECL2)	-	14.7
Replica 8i	7.8	No	No (Meta-binding site on ECL2)	-	13.8
Replica 9i	8.4	Yes	No (Receptor Vestibule)	No (Adenosine unbinding)	7.9
Replica 10i	45.6	Yes	No (Receptor Vestibule)	Yes	0.3

## Supplementary Materials

The complete analysis of SuMD trajectories is available online at https://www.mdpi.com/1420-3049/24/15/2752/s1. Video 1, 2 and 3 are available online at https://zenodo.org/record/3243325#.XT7AW0HdNPY.

## Abbreviations

The following abbreviations are used in this manuscript:

SuMD	Supervised Molecular Dynamics
GPCR	G protein-coupled receptor
A_2A_ AR	A_2A_ Adenosine Receptor
TM	Transmembrane
EC	Extracellular
PDB	Protein Data Bank
MD	Molecular Dynamics
RMSD	Root-mean-square deviation
TMD	Transmembrane domain
ECL	Extracellular loop
POPC	1-palmitoyl-2oleyl-sn-glycerol-3-phospho-choline
OPM	Orientations of Proteins in Membrane
NPT	Isothermal–isobaric ensemble
