# Supplementary material for: Revisiting the Allosteric Regulation of Sodium Cation on the Binding of Adenosine at the Human A2A Adenosine Receptor: Insights from Supervised Molecular Dynamics (SuMD) Simulations
Source: Molecules. 2019 Jul 29;24(15):2752. doi: 10.3390/molecules24152752 (PMC6695830; doi:10.3390/molecules24152752)
Supplement: Supplementary file 1 [file molecules-24-02752-s001.pdf]

Supplementary Materials

Revisiting the Allosteric Regulation of Sodium Cation on the Binding of Adenosine at the Human A_{2A} Adenosine Receptor: Insights from Supervised Molecular Dynamics (SuMD) Simulations

Maicol Bissaro [1], Giovanni Bolcato [1], Giuseppe Deganutti[1,2], Mattia Sturlese[1] and Stefano Moro [1,*]

[1] Molecular Modeling Section (MMS), Department of Pharmaceutical and Pharmacological Sciences, University of Padova, via Marzolo 5, 35131 Padova, Italy

[2] School of Biological Sciences, University of Essex, Wivenhoe Park, Colchester, CO4 3SQ, UK

* Correspondence: stefano.moro@unipd.it; Tel.: +39-049-827-5704

Table of content

Figure S1	SuMD simulation of Na^+ on inactive state of A_{2A} AR	Page 3
Figure S2	SuMD simulation of Na^+ on intermediate-active state of A_{2A} AR	Page 4
Figure S3	ADN locked on ECL2 meta-binding site (intermediate-active state of A_{2A} receptor)	Page 5
Figure S4	ADN reaching canonical conformation on orthosteric binding site (intermediate-active state of A_{2A} AR)	Page 6
Figure S5	ADN locked on ECL2 meta-binding site (inactive state of A_{2A} AR)	Page 7
Figure S6	ADN locked on extracellular vestibule (inactive state of A_{2A} AR)	Page 8
Figure S7	ADN exploring the canonical conformation on orthosteric binding site (inactive state of A_{2A} AR)	Page 9
Figure S8	Sodium binding pathway comparison	Page 10
Figure S9	Sodium unbinding from A_{2A} AR (RMSD)	Page 10
Figure S10	Crystal structures binding site comparison	Page 11
Video 1	Sodium binding pathway on inactive state of A_{2A} AR	Page 12
Video 2	Adenosine different binding pathways collection on the two relevant states of A_{2A} AR	Page 12
Video 3	Adenosine binding pathway on intermediate-active state of A_{2A} AR	Page 12

SuMD replicas have been extensively analyzed through a proprietary tool, able to perform in a fully automated way a series of geometric and energetic analysis of the trajectories. Results for representative simulations are herein reported (grouped in panel).

SuMD final state	Representative replica	Figure number
Na^+ on inactive state	Replica 2	S1
Na^+ on intermediate-active state	Replica 1	S2
ADN locked on ECL2 (intermediate-active state)	Replica 6a	S3
ADN on orthosteric site (intermediate-active state)	Replica 3a	S4
ADN locked on ECL2 (inactive state)	Replica 3i	S5
ADN locked on vestibule (inactive state)	Replica 5i	S6
ADN on orthosteric site (inactive state)	Replica 10i	S7

In detail, for each representative replica six graphs are reported, summarizing:

A. The RMSD of ligands (sodium or adenosine), computed with respect to crystallographic reference (4EIY or 2YDO).

B. The RMSD computed on protein Cα atoms.

C. The ligand-protein interaction energy (kcal/mol), defined as the sum of the electrostatic and vdW components, calculated on the basis of the force field (FF) terms.

D. Per-residue decomposition of ligand-protein interaction energy (kcal/mol), with the aim to quantitatively characterize the role played by different protein residues during molecular recognition.

E. The time (ns) evolution of the per-residue ligand-protein interaction energy (kcal/mol).

F. An indication about the residues mostly involved in the binding process, for each target residue.

G. The total number of contacts between the ligand and the nearest protein residues (4 Å) during SuMD simulation, to obtain an indication about the residues mostly involved in the binding process.

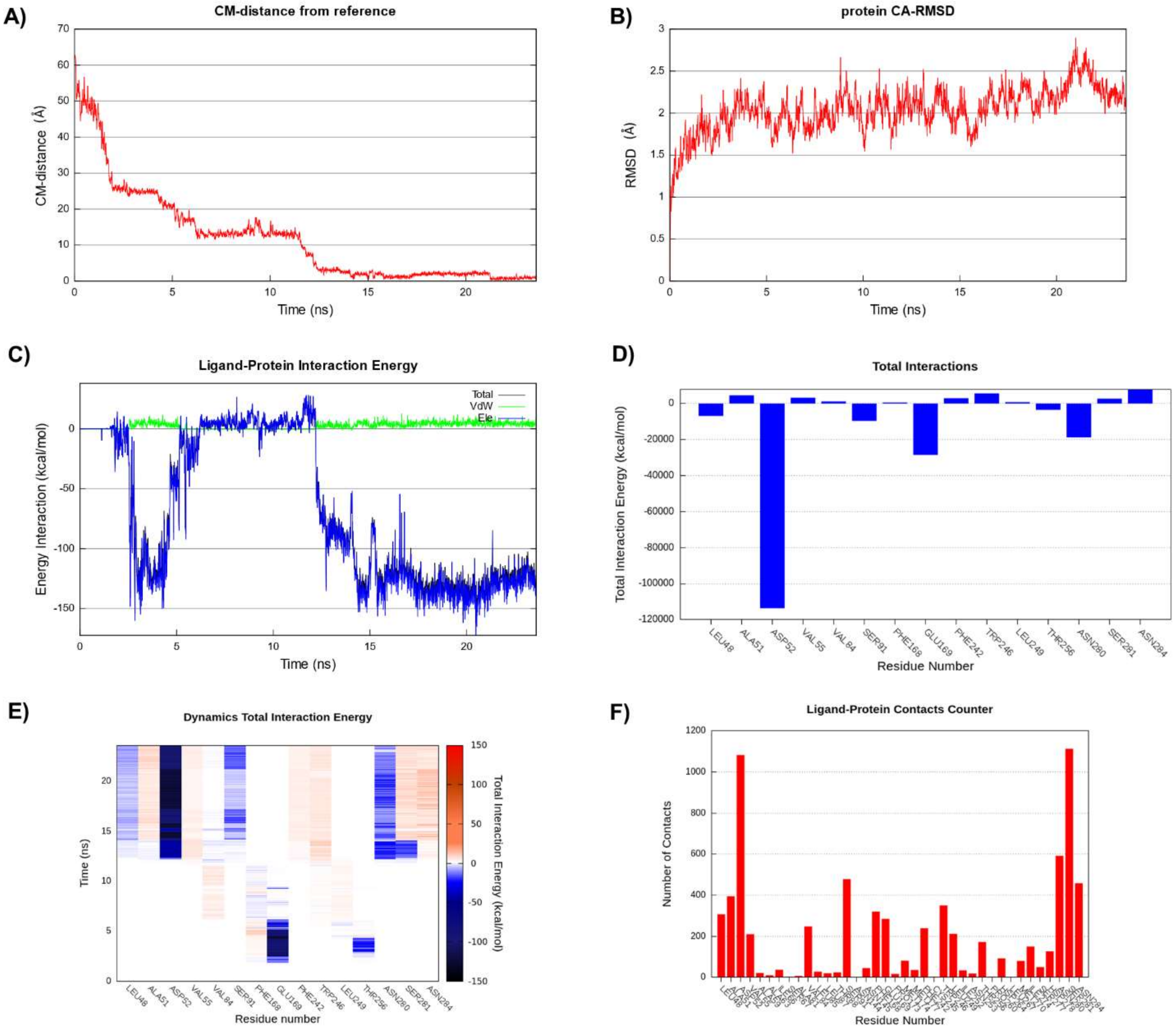

Figure S1 SuMD simulation of Na^+ on inactive state of A_{2A} AR.

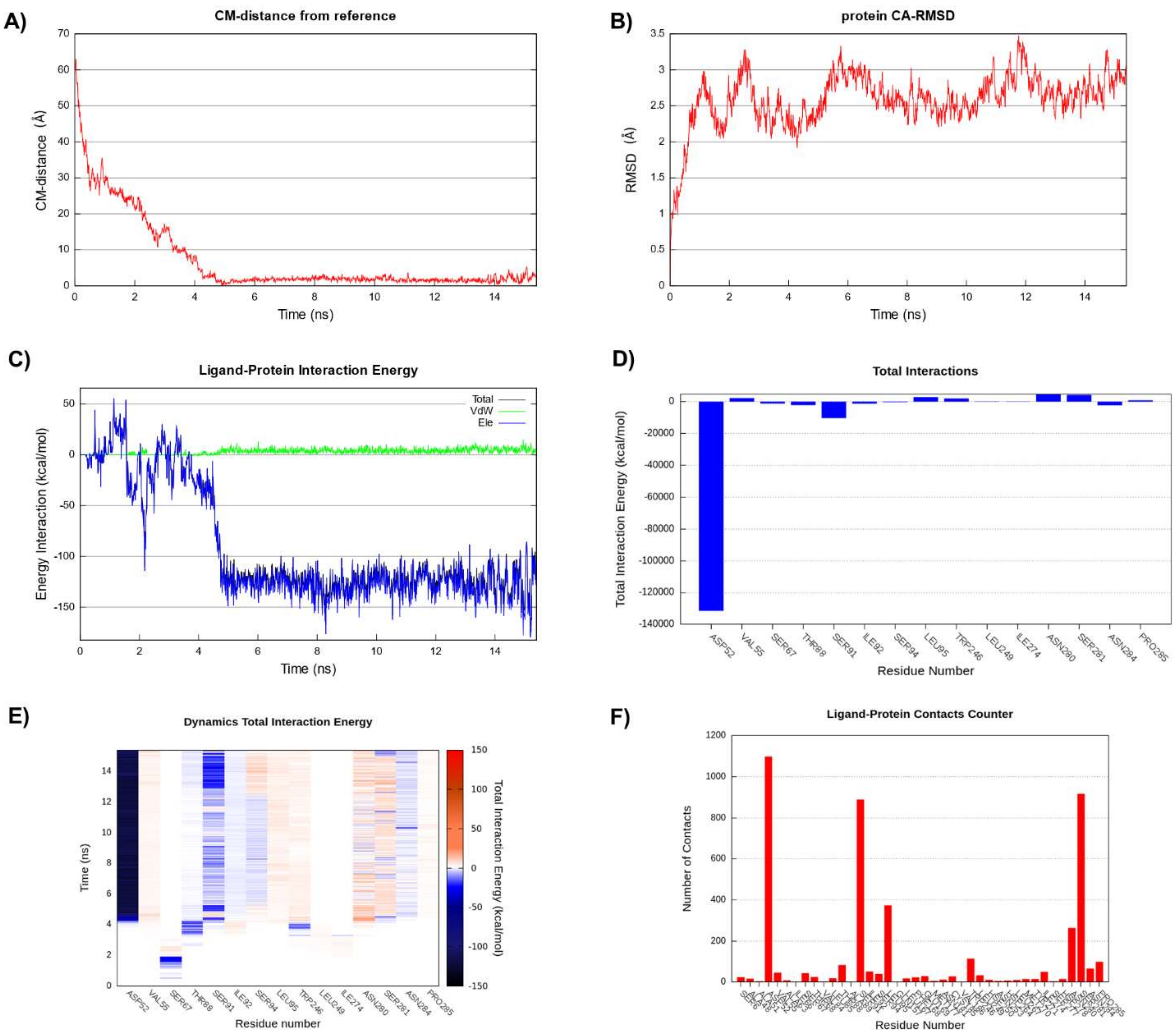

Figure S2 SuMD simulation of Na^+ on intermediate-active state of A_{2A} AR.

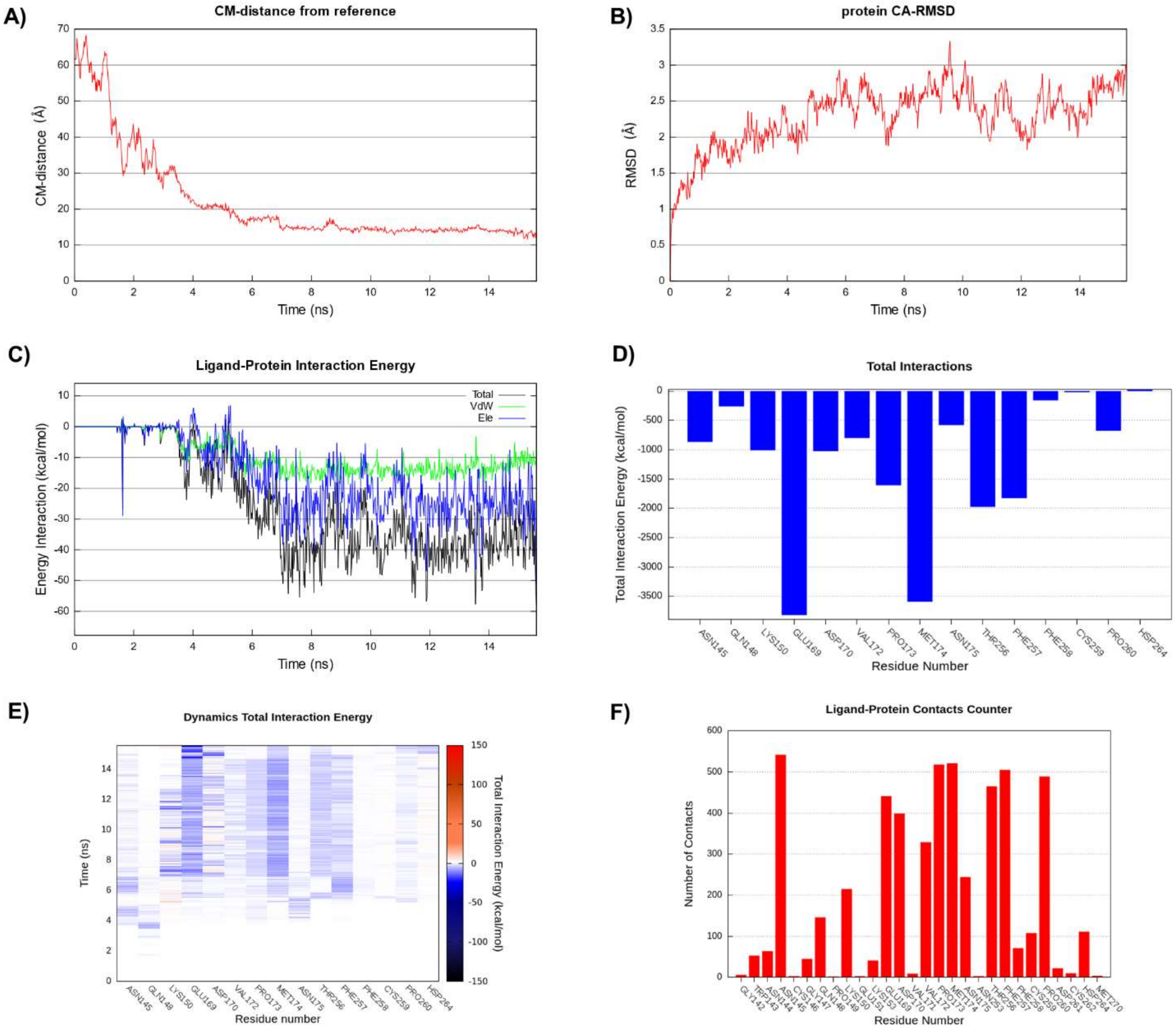

Figure S3 ADN locked on ECL2 meta-binding site (intermediate-active state of A_{2A} receptor).

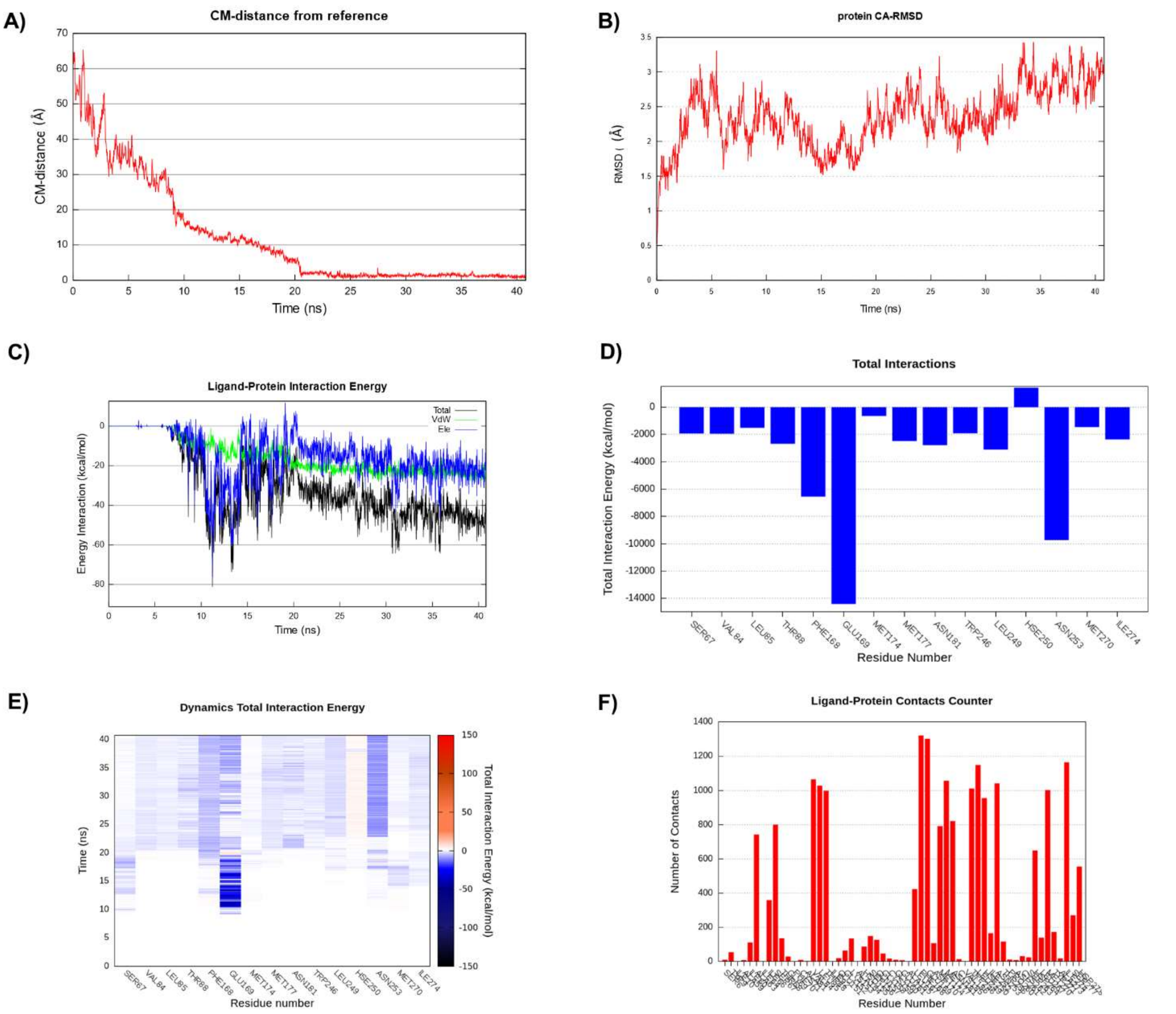

Figure S4 ADN reaching canonical conformation on orthosteric binding site (intermediate-active state of A_{2A} AR).

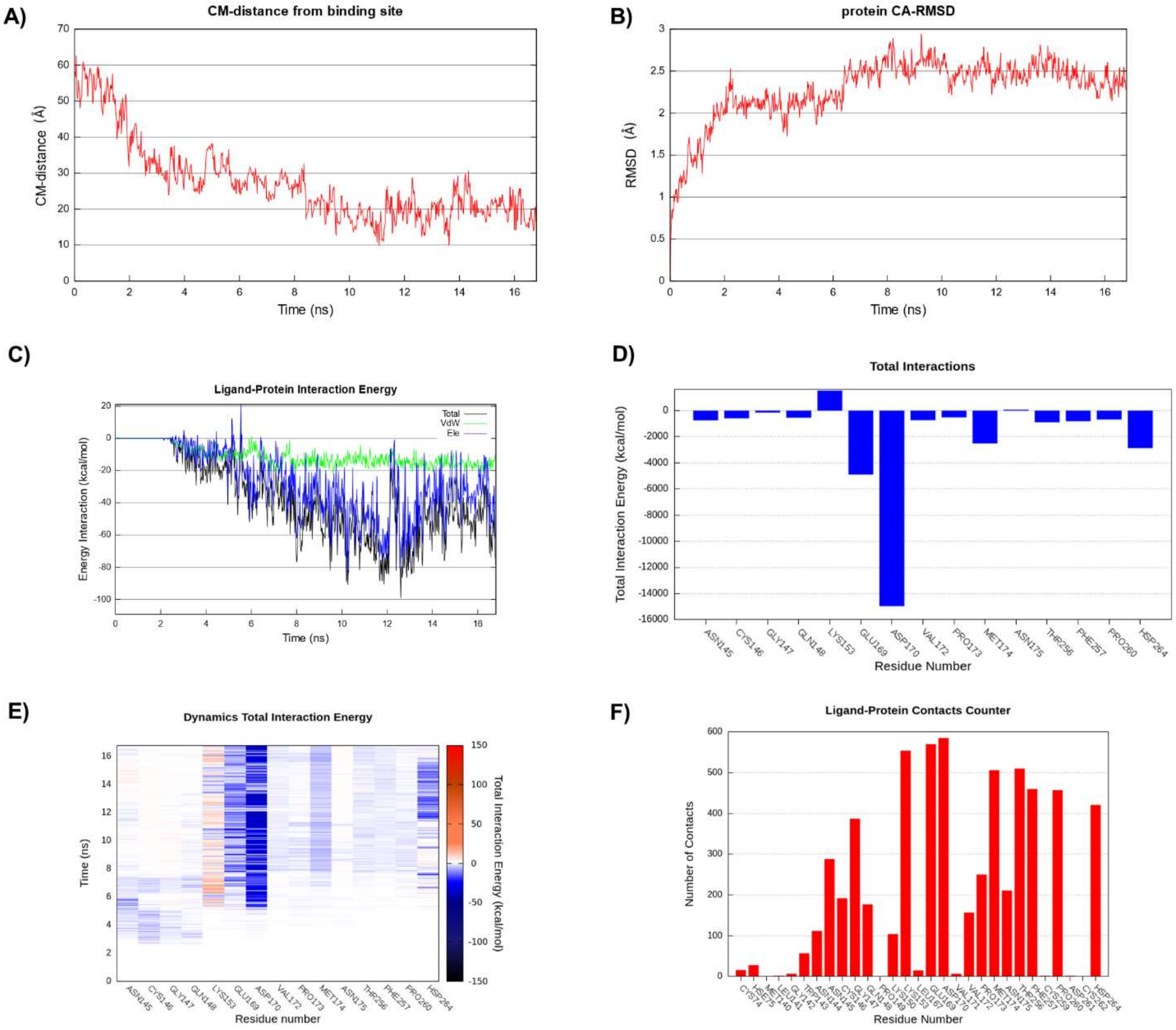

Figure S5 ADN locked on ECL2 meta-binding site (inactive state of A_{2A} AR).

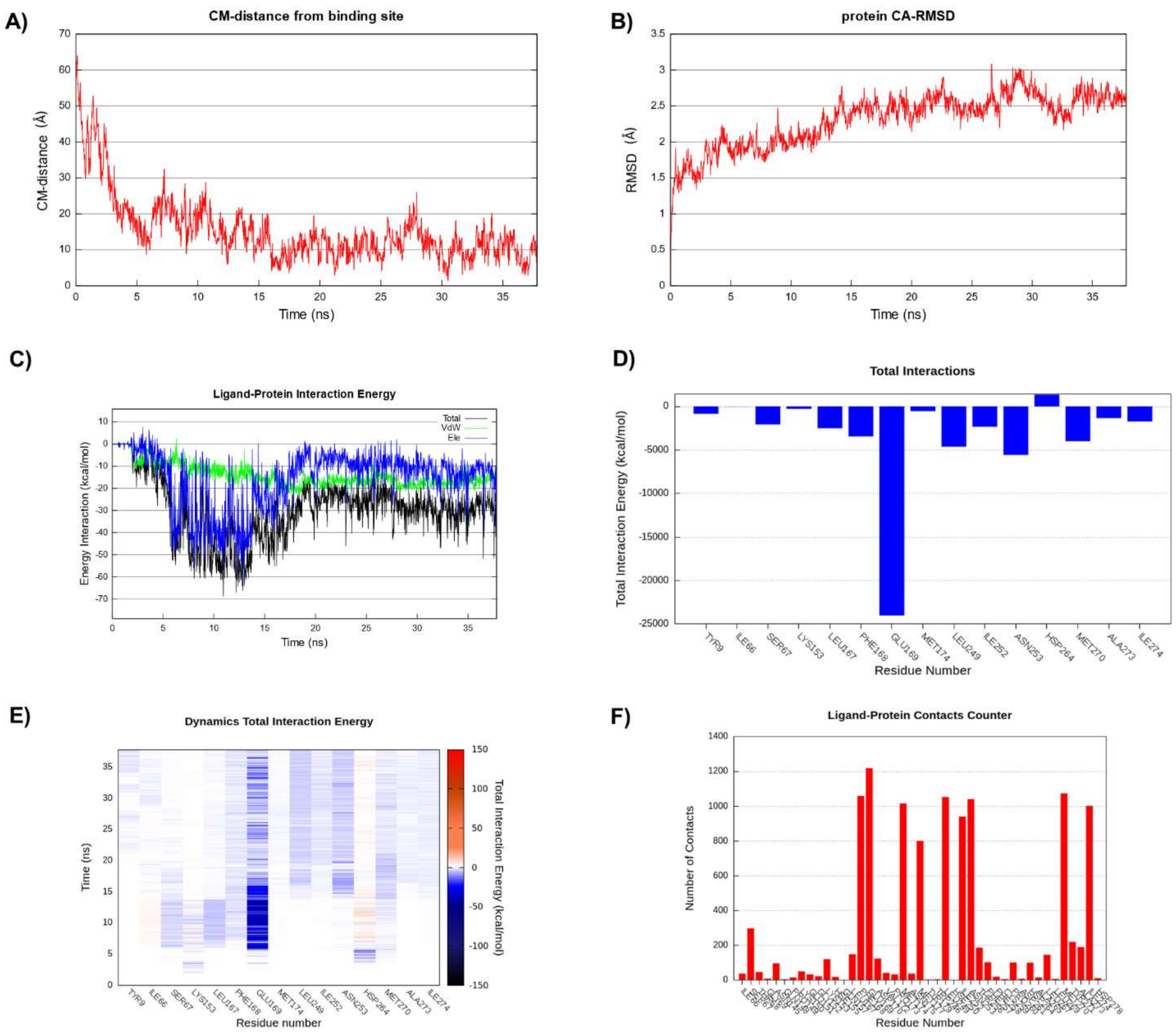

Figure S6 ADN locked on extracellular vestibule (inactive state of A_{2A} AR).

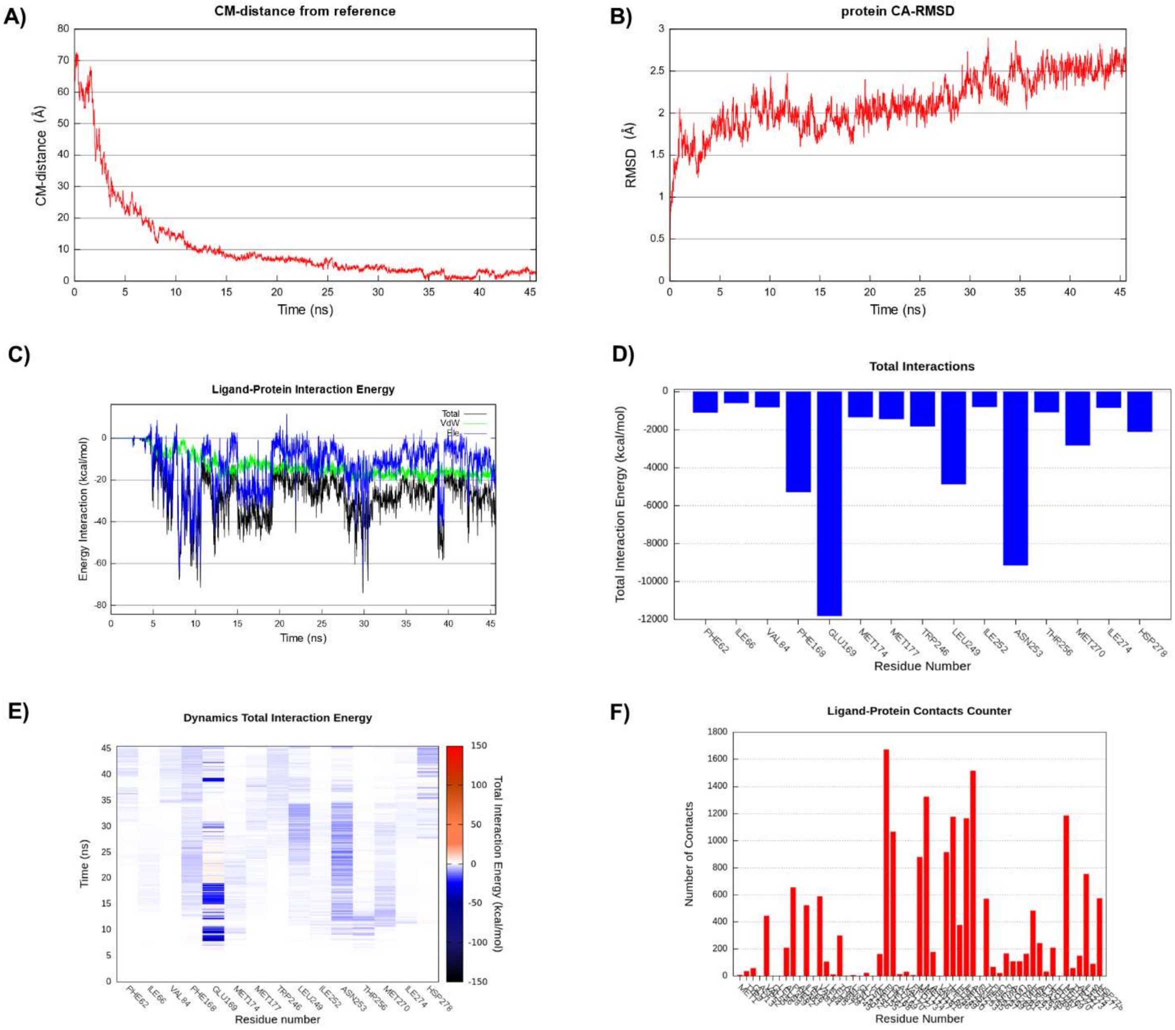

Figure S7 ADN exploring the canonical conformation on orthosteric binding site (inactive state of A_{2A} AR).

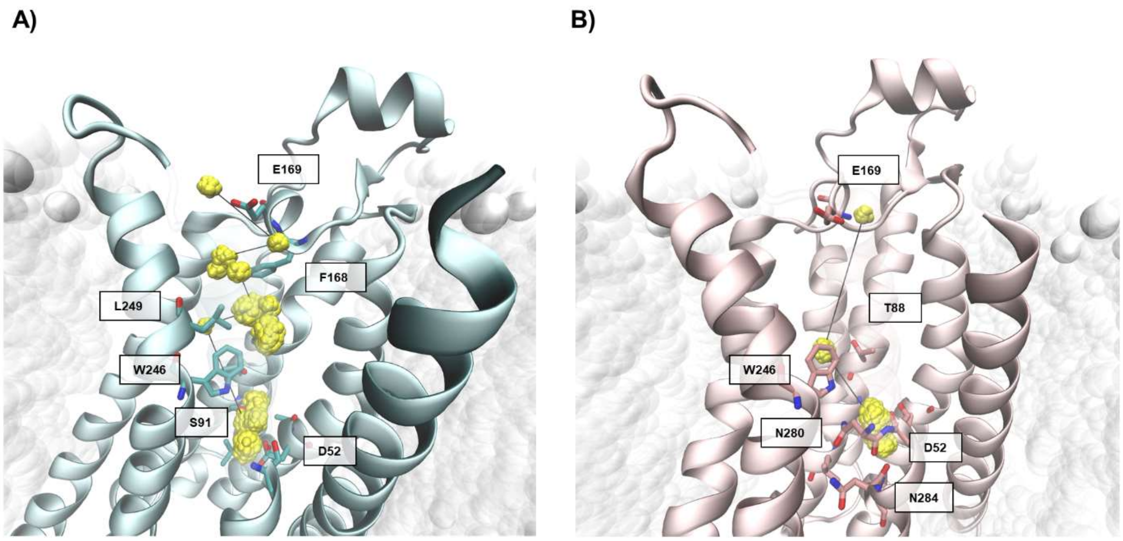

Figure S8 Sodium binding pathway comparison: To map the regions of the receptor in which sodium ion is stationed more frequently during its approach to the allosteric site**,** SuMD trajectories were geometrically clustered. On the Panel A ion binding pathway, along with most contacted A_{2A} AR in its inactive like state are depicted. On the right side, panel B represent the sodium recognition mechanism on the other relevant receptor conformation, the one able to recognize agonist molecules.

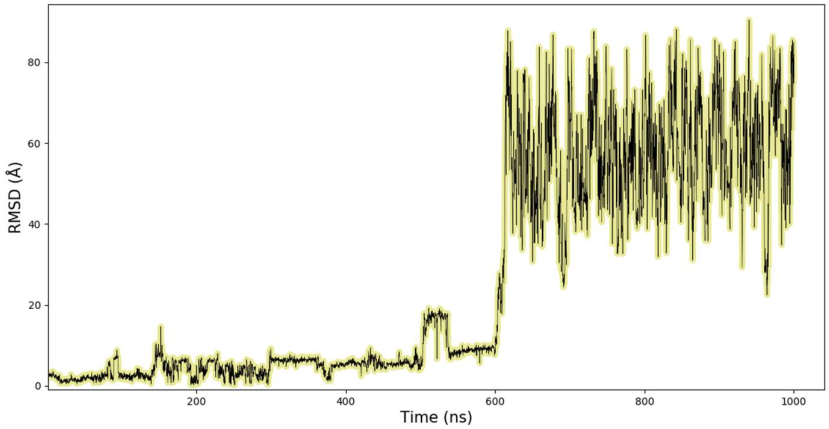

S9 Sodium unbinding from A_{2A} AR (RMSD): to verify the reversibility of the sodium ion recognition process, starting from the coordinated structures previously obtained through the SuMD trajectories, an unbiased MD simulation was performed. The graph reports the RMSD of Na^+ atomic coordinates with respect to crystallographic reference (4EIY). 600 ns of simulation time were sufficient to sample a spontaneous ion unbinding event from the allosteric site toward the extracellular environment.

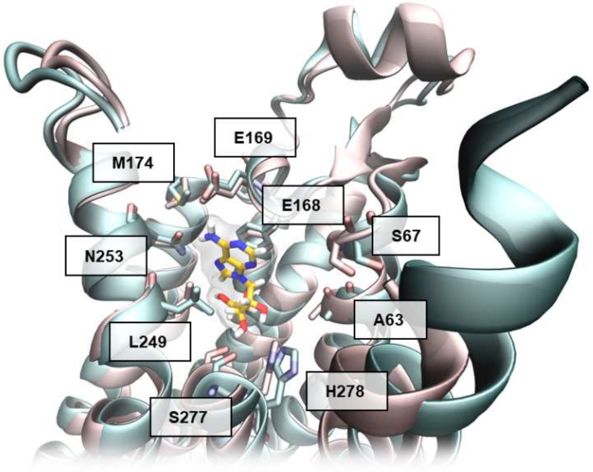

RMSD of binding site residues: 1.14 Å

S10 Crystal structures binding site comparison: comparison between residues composing the orthosteric binding site of the two pharmacologically relevant A_{2A} AR states, the one intermediate-active like (2YDO) and the inactive (4EIY). Image shows how the overall organization is quite conserved, as confirm by modest RMSD value of 1,14 Å, computed on aforementioned residues.

SuMD trajectories Videos

Video 1: Sodium binding pathway on inactive state of A_{2A} AR.

The video is composed of four synchronized and animated panels that depict the molecular trajectory obtained by the SuMD simulation considering different aspects of the simulation. The time evolution is reported in a nanosecond. In the first panel (upper-left), the molecular representation of the macromolecular system is shown. The A_{2A} AR inactive state backbone is represented by the ribbon style (cyan colour) and the residues within 4 Å of sodium ion during entire simulation are dynamically shown. Na^+ is rendered showing its VdW volume in yellow. In the second panel (upper-right), the dynamic distance of sodium center of mass (CM) from the A_{2A} AR allosteric binding site during the trajectory is reported. In the third panel (lower-left), the MMGBSA energy profile is reported. The animated red circle highlights the value of the corresponding frame. The trend is depicted by a continuous black line obtained by smoothing the raw data (grey circles) using a Bezier curve procedure. In the fourth panel (lower-right) cumulative electrostatic interactions are reported for the 15 A_{2A} AR residues most contacted by sodium during the whole simulation.

Video 2: Adenosine different binding pathways collection on the two relevant states of A_{2A} AR

The video is composed by two panel, which summarize the recognition process of the adenosine agonist, sampled by means of the supervised molecular dynamics methodology, in the two pharmacologically relevant states of the receptor. In particular, on the right side are shown simultaneously all ten replicas collected starting from the intermediate-active conformation of the A_{2A} AR (pink ribbon). The meta-binding site located at the level of the ECL2 and the orthosteric binding site were highlighted. On the left side are represented simultaneously all ten replicas collected starting from the inactive conformation of the A_{2A} AR (cyan ribbon). The meta-binding site located at the level of the ECL2 and the extracellular receptor vestibule were highlighted.

Video 3: Adenosine binding pathway on intermediate-active state of A_{2A} AR.

The video is composed of four synchronized and animated panels that depict the molecular trajectory obtained by the SuMD simulation considering different aspects of the simulation. The time evolution is reported in a nanosecond. In the first panel (upper-left), the molecular representation of the macromolecular system is shown. The A_{2A} AR intermediate-active state backbone is represented by the ribbon style (pink colour) and the residues within 4 Å of sodium ion during entire simulation are dynamically shown. Adenosine molecule is rendered by orange carbon atoms and by a transparent surface. In the second panel (upper-right), the dynamic distance of agonist center of mass (CM) from the A_{2A} AR allosteric binding site during the trajectory is reported. In the third panel (lower-left), the MMGBSA energy profile is reported. The animated red circle highlights the value of the corresponding frame. The trend is depicted by a continuous black line obtained by smoothing the raw data (grey circles) using a Bezier curve procedure. In the fourth panel (lower-right) cumulative electrostatic interactions are reported for the 15 A_{2A} AR residues most contacted by adenosine during the whole simulation.
